# Square Membrane Resonators Supporting Degenerate Modes of Vibration for High-Throughput Mass Spectrometry of Single Bacterial Cells

**DOI:** 10.1021/acssensors.3c00338

**Published:** 2023-05-01

**Authors:** Adrián Sanz-Jiménez, Jose J. Ruz, Eduardo Gil-Santos, Oscar Malvar, Sergio García-López, Priscila M. Kosaka, Álvaro Cano, Montserrat Calleja, Javier Tamayo

**Affiliations:** Bionanomechanics Lab, Instituto de Micro y Nanotecnología, IMN-CNM (CSIC), Isaac Newton 8 (PTM), Tres Cantos, E-28760 Madrid, Spain

**Keywords:** nanomechanical spectrometry, membrane sensors, biomechanical sensors, degenerate modes, mass sensors, single-cell mass spectrometry

## Abstract

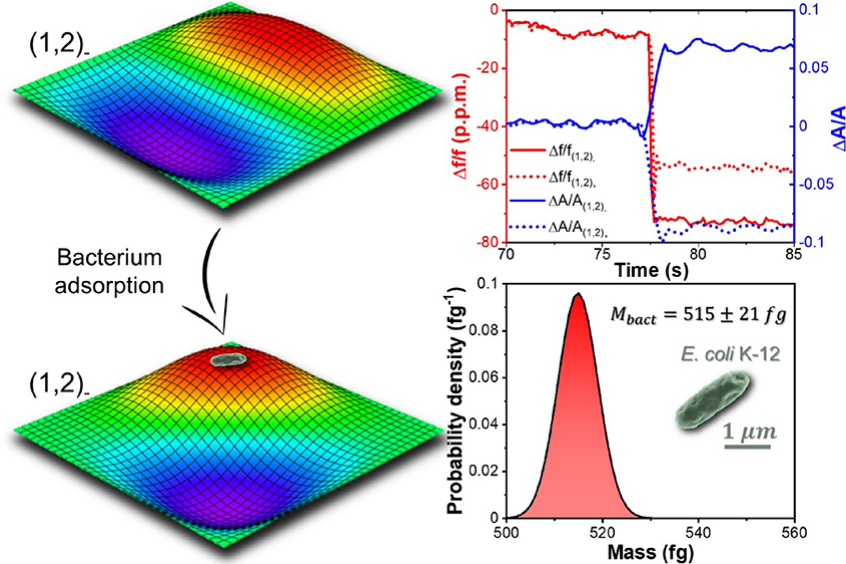

In nanomechanical mass spectrometry, sensing devices are commonly placed in the vacuum environment and a stream of analytes is directed toward the sensor surface for measurement. Beam structures, such as double-clamped nanobeams and nanocantilevers, are commonly used due to their low inertial mass and the simplicity of the analytical models for mass extraction. The drawback of such structures is their low capture areas, compromising the capture efficiency and throughput of this technique. Bi-axisymmetric resonators, such as ultrathin square or circular membranes, arise as an optimal geometry to maximize capture efficiency while minimizing the device inertial mass. However, these structures present degenerate mechanical modes, whose frequency perturbations upon analyte adsorption are not well described by commonly used models. Furthermore, prior knowledge of the vibration mode shapes of the sensor is crucial for the correct calculation of the analyte’s mass, and the mode shape of degenerate modes may change significantly after every adsorption event. In this work, we present an accurate analytical theory to describe the effect of mass adsorption on the degenerate modes of square membrane resonators and propose two different methods based on the new theory to update the vibration mode shapes after every adsorption event. Finally, we illustrate the problem experimentally obtaining the mass and adsorption position of individual *Escherichia coli* K-12 bacterial cells on commercial square silicon nitride membranes fabricated with very small tolerances.

Nanomechanical resonators play a fundamental role for the development of new techniques and technologies for very different applications, ranging from quantum measurements to chemical or biological sensing.^1−3^ Due to their high dynamic range and unprecedented sensitivity, one of the most popular applications of nanomechanical resonators is mass sensing.^4−6^ The principle is very simple; when a small particle accretes to the sensor surface, the resonance frequency of the resonator changes due to the modification of the kinetic energy of the system. This change depends on the mass ratio between the particle and the resonator and also on the adsorption position.^7^ If the adsorption position is not known, several modes of vibration must be measured to retrieve the mass and position from the changes in the resonance frequencies, which is known as multimode nanomechanical mass sensing.^8^ Interestingly, multimode nanomechanical sensing allows not only to obtain the mass of analytes but also the stiffness and even the shape.^9−12^ It has been used also to measure the mass of large particles when the mass ratio is so high that the mode shapes associated to the resonance frequencies are altered due the presence of the particle.^13,14^ All these features of nanomechanical resonators have led to the development of a new type of mass spectrometry called nanomechanical mass spectrometry,^15^ which basically uses nanomechanical resonators as sensor elements, and the mechanical properties of the analytes for the identification. This technique has proven to be extremely useful for the detection, mechanical characterization, and identification of biological particles such as proteins, bacteria, or viruses.^16−18^ Importantly, nanomechanical mass spectrometry does not require the fragmentation of the particles, neither their ionization,^19^ and can perform well even at atmospheric pressure,^20^ enabling the characterization of biological entities on their intact conformations, in contrast with conventional mass spectrometry (MS). In addition, conventional MS struggles when applied for relatively heavy particles, such as bacteria or viruses, due to the big uncertainty in the determination of the mass that comes from the large number of charge states^21^ that is attributed to incomplete desolvation of these big particles.^22^ Conventional MS has reached an experimental mass limit of 18 MDa.^23^ Notably, researchers suggest that this technology has an intrinsic mass limit of 20 MDa^24^ in homogeneous samples, being much lower for heterogeneous ones.^25^ Recently, charge detection mass spectrometry (CDMS) has emerged as a novel and very promising technology.^26,27^ CDMS can simultaneously measure the mass-to-charge ratio and the charge of single particles in the MDa regime with extremely high sensitivity.^28^ CDMS has characterized the mass of intact exosome particles^29^ up to 90 MDa and has reached the GDa regime with anisotropic composite nanoparticles.^30^ However, this limit is still far from biological analytes like bacterial cells in the hundreds of GDa, where nanomechanical MS can play a crucial role.

One of the most important challenges that nanomechanical MS faces is to deliver the analytes from the sample (that can be a liquid, a surface, or simply the air in a room) to the sensor surface efficiently. The loss of particles in the transportation itself and the tiny capture area that nanomechanical resonators usually provide are the main limitations for capture efficiency. To improve the capture efficiency, researchers have worked extensively on two complementary aspects. On one hand, they have pushed toward focusing the particle beam in the smallest cross section possible, developing systems based on the use of aerodynamic lenses. Even though these systems enable reducing the particle beam size, the covered areas are still relatively large,^18,19,31−33^ being above 1 mm^2^. Another approach is the use of self-focusing systems,^20^ which has achieved very promising results, reducing the particle beam size to areas of about 200 μm^2^. On the other hand, researchers have proposed the use of arrays of individual or coupled resonators.^18,34^ However, this approach requires extremely sophisticated readout mechanisms or complex data interpretation. Another solution is to optimize the size and shape of the resonator to maximize the covered area of the cross section of the particle beam. In this regard, if the particle beam is symmetric (a circular beam is the most common), the optimum shape of the resonator should have an aspect ratio close to one, such as circular or square, covering as much area as possible.^35^ At the same time, the thickness of the resonator must be as low as possible to not compromise the mass sensitivity. Another important aspect of nanomechanical MS is that it requires tracking the frequencies of several mechanical modes to extract the adsorption position and mechanical properties of the particles. Moreover, it is also required to accurately know the mode shapes associated to the tracked frequencies prior to analyte adsorption. Therefore, it is necessary that the mode shapes remain the same during a whole measurement or, in the case they change, to track the changes and update the new mode shapes before the next event occurs. Note that this condition is usually taken for granted, as it is well satisfied for structures like cantilevers or double-clamped beams, the two most popular geometries used in the field. However, as we have emphasized, these geometries are not the best ones regarding the improvement of capture efficiency. On the other hand, structures with higher capture efficiency, like circle or square membranes, support degenerate modes, i.e., orthogonal eigenmodes that vibrate at exactly the same frequency.^36^ In this case, the mode shape can be any linear combination of the mode shapes of the same structure, considering no degeneration. Notably, when two modes are degenerate, they are extremely sensitive and any small perturbation could break the degeneration, thus making a particular linear combination of the modes energetically more favorable than the rest.^37^ In practice, for experimental devices, due to the limit of resolution in the fabrication process, there is always a particular linear combination that is energetically more favorable, leading to the degeneration breakage of the sensor modes. We clarify that in this situation, even if the frequencies are not exactly the same, a small perturbation can still cause big changes in the eigenmode shapes if the energy involved is of the same order of the small energy difference between the modes. In the particular case of mass sensing applications, as in nanomechanical MS, the adsorption of a small particle can induce important changes in the mode shapes and thus commonly used models that assume that the mode shapes remain unaltered after analyte adsorption are not valid. In this work, we present a precise theory that describes the effect of particle adsorption on the eigenfrequencies and mode shapes of the degenerate modes supported by square membrane resonators. Notably, the theory can be easily extended to any other geometry that supports degenerate modes of vibration, such as circular membranes. Then, based on the new theory, we propose and compare two different methods to calculate the change of the mode shapes upon analyte adsorption. These methods will allow precise calculation of the mass of the analytes as they accrete sequentially on the membrane surface in a nanomechanical MS experiment.

## Analytical Theory

Let us consider a rectangular membrane of length *L_x_*, width *L_y_*, and thickness *h* that is centered at the origin of the coordinate system. We define the aspect ratio of the membrane as . A membrane can be considered as a 2D analogy of a double-clamped beam. Depending on the residual stress, the resonator can be in a beam state, a string state, or a hybrid between these two.^38^ In this work, we consider the case of a stressed membrane where the bending energy can be neglected, which is the most common case found in practice. For such a membrane, the different eigenfrequencies associated to the out-of-plane vibrations can be expressed as a function of two natural numbers (*m*, *n*) as^35^
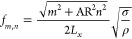
1where σ and ρ are the stress and density of the membrane, respectively. The mode shapes associated to these eigenfrequencies are given by
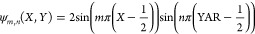
2where *X* = *x*/*L_x_* and *Y* = *y*/*L_x_* are the coordinates normalized to the length of the membrane. Interestingly, for *m* ≠ *n*, the eigenfrequencies *f*_*m*, *n*_ and *f*_*n*, *m*_ can be very close if the aspect ratio is sufficiently close to 1. In fact, they are exactly the same for a perfectly square membrane (AR = 1), which is the case of pure degeneration. As we mentioned before, for a perfectly square membrane, the mode of vibration associated to the pair (*m*, *n*) can be any linear combination of the two modes ψ_*m*, *n*_(*X*, *Y*) and ψ_*n*, *m*_(*X*, *Y*). We then propose a solution for the vertical displacement that is given by
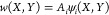
3where the index *i* can take values 1 and 2, *A_i_* are arbitrary amplitudes, ψ_1_(*X*, *Y*) = ψ_*m*, *n*_(*X*, *Y*) and ψ_2_(*X*, *Y*) = ψ_*n*, *m*_(*X*, *Y*), and the Einstein’s summation notation of repeated indices is being used. As a convention, we assume that *m* < *n* and therefore the frequencies before adsorption satisfy that *f*_*m*, *n*_ ≥ *f*_*n*, *m*_. After the adsorption of an analyte of mass *m_a_* at position (*X*_0_, *Y*_0_), the total kinetic and potential energies of the system can be expressed as
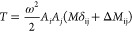
4
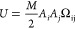
5where ω is the angular frequency of the system, *M* is the total mass of the membrane, δ_*ij*_ is the Kronecker delta, Ω_*ij*_ is a diagonal matrix whose elements are ω_*m*, *n*_^2^ and ω_*n*, *m*_^2^, and Δ*M_ij_* represents the added mass matrix that is given by
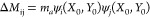
6

Note that we assume that the potential energy of the membrane is not altered upon analyte adsorption, which is a very good approximation for the case of stressed membranes.^35^ Applying the Rayleigh–Ritz principle, the total kinetic energy must be equal to the total potential energy, giving as a result the equation for the system
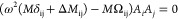
7

The only way that eq 7 has a solution different than the trivial solution is making the determinant of the system equal to zero,
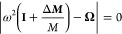
8where **I** is the unity matrix. Equation 8 is the new eigenfrequency equation whose solutions are the new eigenfrequencies that are given by
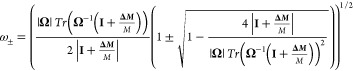
9where *Tr* represents the trace and the + and – signs refer to the fast and slow modes, respectively. The new mode shapes associated to these eigenfrequencies can be found, applying a geometric rotation θ to the vector formed by the two eigenmodes ψ_*m*, *n*_ and ψ_*n*, *m*_,
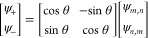
10

This rotation θ is the one that makes the matrix of the system (eq 7) diagonal and can be expressed as
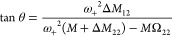
11

Equations 10–11 show how the mode shapes change when small particles are adsorbed on the membrane surface. This change depends on the position of the adsorbed particle, and it increases as the aspect ratio of the resonator is closer to 1, as well as when the analyte to sensor mass ratio increases. Note that this formalism is valid even if the mode shapes of the membrane before adsorption are not ψ_*m*, *n*_ and ψ_*n*, *m*_ but two linear combinations of them ψ_+0_ and ψ_–0_, as it can occur in an experiment with multiple adsorptions. In that case, the pair (ψ_*m*, *n*_, ψ_*n*, *m*_) must be replaced by (ψ_+0_, ψ_–0_), and the frequencies (ω_*m*, *n*_, ω_*n*, *m*_) by (ω_+0_, ω_–0_). From eq 9, we can calculate the relative frequency shift due to the adsorption for each of the eigenfrequencies. Assuming that the mass of the analyte is much smaller than the mass of the membrane, we can express the relative frequency shifts as
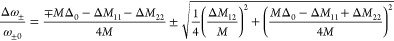
12where Δ_0_ is the relative separation of the square of the frequencies before adsorption . Note that as the aspect ratio increases from 1, the separation between the two resonance frequencies becomes larger (Δ_0_ increases). In the limit of  and , eq 12 reduces to the classical expression for not degenerate modes 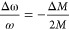
13 and the rotation angle θ will be negligible. Interestingly, eq 12 reduces as well to the classical eq 13 when Δ*M*_12_ = 0 even if the difference between frequencies is small. This can occur when the analyte adsorbs at a position where there is a node of the fast mode before adsorption. In this case, as eq 11 states, there will be no change in the mode shapes. This situation can also occur if the adsorption takes place at a position where there is a node on the slow mode. However, if the particle is massive enough, it might produce a change in θ of π/2 (modes crossing), and in this case, eq 13 must be slightly changed, accounting for the initial frequency difference Δ_0_.

### Validation of the Theory with Finite Element Simulations

**Figure 1:**
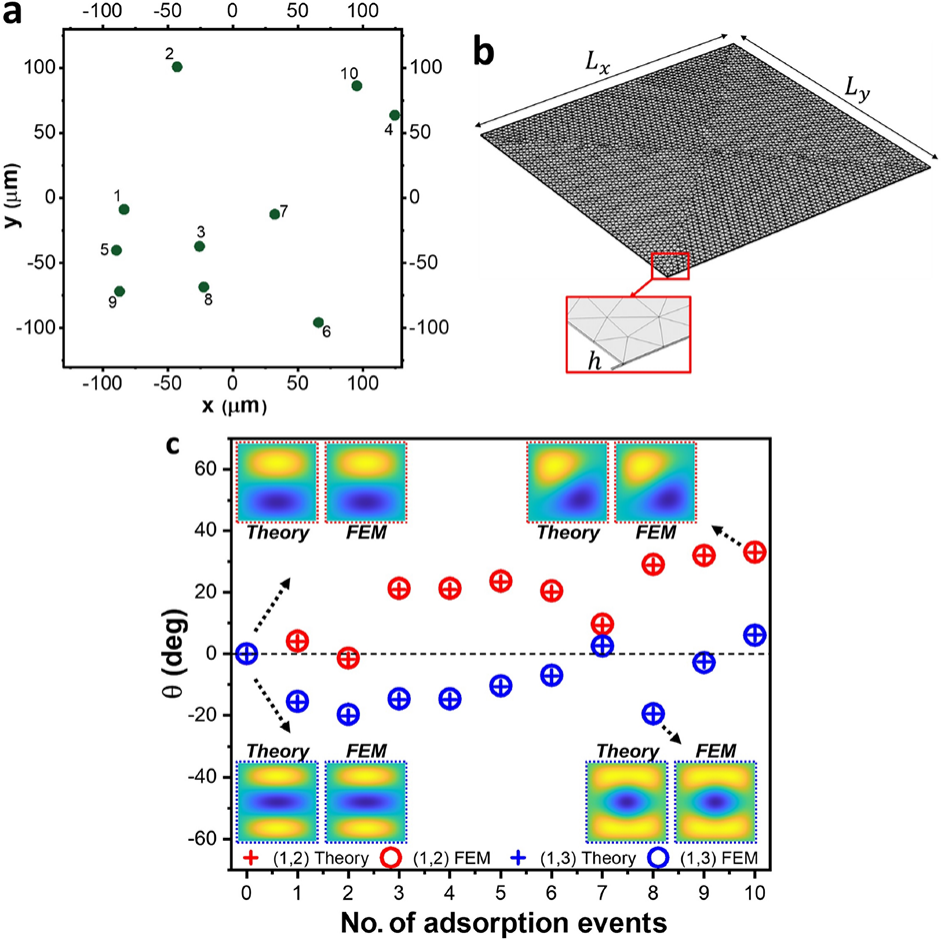
Comparison of the theoretical model with FEM simulations. (a) Positions (green circles) and order (labeled numbers) of the 10 randomly distributed adsorbed particles. (b) Image of the simulated membrane sensor showing the mesh distribution and dimensions. (c) Comparison between the values of the parameter θ obtained by FEM (open circles) and those predicted by the new theory (crosses) for the pair of modes (1,2) (red) and (1,3) (blue). The insets show a colormap of the mode shape obtained by FEM and predicted by the new theory right after some of the adsorptions for the fast mode. The excellent agreement between FEM and theory validates the new model.

To validate the theory, we performed finite element method (FEM) simulations of 10 randomly distributed mass adsorptions (Figure 1a) in a square silicon nitride membrane sensor of 50 nm thickness, 250 μm width, an aspect ratio of 1.0001, and a total mass of 9.6875 ng (Figure 1b). A pre-stress of 212 MPa is applied to the membrane before the eigenfrequency analysis.

The adsorbed masses were considered as point-like particles with a mass of 500 fg each, which could be the case of an *Escherichia coli* bacterium. It is important to note that the theory assumes that there are no changes in the mode shapes other than those coming strictly from degeneration effects. If the ratio between the mass of the particle and the mass of the membrane is very high, there might be other effects on the mode shapes that do not have to do with degeneration.^13,14^ Simulations for higher mass ratios and the range of validity of the theory are available in the Supporting Information Section S1. We focus on the study of the degenerate pairs of modes (*m*, *n*) = (1,2) and (*m*, *n*) = (1,3). Figure 1c compares the parameter θ and the normalized frequencies obtained by the simulations with those predicted by the developed theory after each adsorption. The parameter θ in the numerical simulation is obtained by fitting the mode shapes to eq 10. The graphs show excellent agreement between the developed theoretical model and the simulations, which confirms the validity of the above-presented theory.

### Methods to Obtain the Parameter θ after Each Adsorption Event

In this section, we will propose two different methods to obtain the parameter θ after each adsorption event in a common multimode nanomechanical MS experiment. For both methods, it is necessary to know the mode shapes of the membrane before the experiment starts. The first method is based on eq 11. If we know the frequencies before and after the adsorption, and the mass and position of the adsorbed particle, we can use eq 11 to update the mode shape for the next event. The calculation of the mass and position of the adsorbed particle can be made by the inverse problem described elsewhere.^35^ The only difference is that for the modes that have degeneration, we must use eq 12 instead of eq 13. As we have mentioned, the mode shapes must be known before the experiment starts, and then, they must be updated after every single event using eq 11 to calculate the mass and position of the next particle correctly. The main advantage of this method is that we can update the mode shapes after every single event without the need of any additional measurements, just using the tracked frequencies that are anyway necessary for the resolution of the inverse problem. However, the main drawback of the method is that the errors in mass and position committed in the resolution of the inverse problem may induce considerable errors in the calculation of θ and these errors will be accumulated as new adsorption events take place.

The second method is based on the measurement of the amplitudes of the frequency peaks by means of the laser beam deflection technique. This technique is commonly used to measure the resonance frequencies of the out-of-plane modes of mechanical structures like cantilevers, doubly clamped beams, or membranes and consists of illuminating the resonator surface with a laser spot and then collecting the reflected beam on a photodetector.^39^ The out-of-plane movement of the resonator produces the movement of the laser spot on the photodetector whose amplitude is proportional to the slope of the mode shape at the position where the laser spot is placed on the resonator surface. For a membrane, after an adsorption event, the relative change in amplitude that we measure in the photodetector is given by
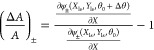
14where (*X*_ls_, *Y*_ls_) is the position of the laser spot on the resonator, θ_0_ is the parameter θ before the adsorption, and Δθ is its variation after the adsorption. Note that we have used the slope along the *x* direction in eq 14 but it would be completely analogous with the slope along the *y* direction. Assuming that the amplitude noise measured with the beam deflection technique is Gaussian, we can form the probability density function of the relative change in amplitude as follows:
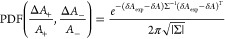
15where  is the vector whose components are the relative change in amplitude measured by the laser beam deflection technique for the fast and slow modes, , and Σ is the covariance matrix of the noise of the relative change in the amplitude of the two pairs of modes. Assuming that we know the position of the laser spot on the resonator and the parameter θ_0_, we can express the probability density (eq 15) as a function only of the variation Δθ, PDF(Δθ). The value of Δθ that maximizes this function will be then the change in θ that we are searching after the particle adsorption. The advantage of this second method is that the error depends only on the noise of the measurement of the relative change of amplitudes, and the calculation is faster than the first method as it does not require the knowledge of the mass and position of the adsorbed particle. On the other hand, it is necessary to track the amplitudes at every time, but this can be easily done with the same optical transduction mechanism.

## Experimental Section

### Adsorption of *E. coli* Bacterial Cells on a Square Silicon Nitride Membrane

**Figure 2:**
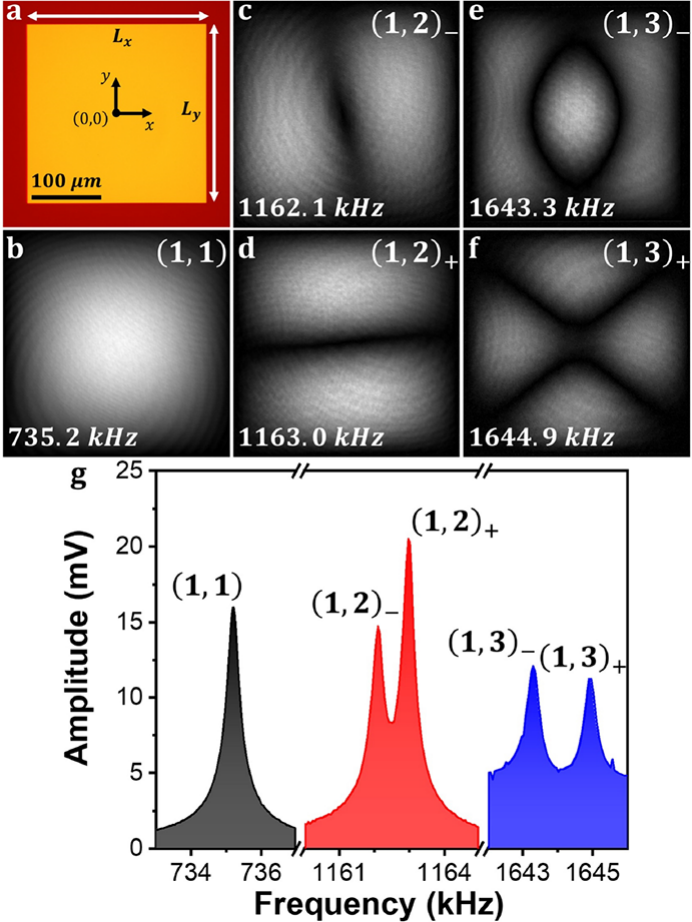
Membrane resonator mechanical modes. (a) Optical microscope image (20× magnification, bright field) of the silicon nitride membrane resonator (yellow) used in this work. The image also shows the 200 μm-thick silicon frame (red). (b–f) (1,1), (1,2)_−_, (1,2)_+_, (1,3)_−_, and (1,3)_+_ vibrational mode shapes of the membrane obtained with a digital holographic microscope (DHM). (g) Measured mechanical spectra of the membrane resonator.

To illustrate the problem experimentally, we have performed an experiment depositing individual cells of *E. coli* K-12 bacteria on the surface of a square silicon nitride membrane resonator by using a nanomechanical spectrometer system. The experiment consists in nebulizing bacteria from a solution by means of the electrospray ionization technique. This nebulization enters the spectrometer and is guided through different vacuum stages to the sensor surface.^35^ Five different eigenfrequencies of the sensor are tracked continuously using the laser beam deflection method (Supporting Information Section S2) so every time a bacterium reaches the sensor surface, we observe abrupt changes in these eigenfrequencies. We also monitor the amplitude of each of the frequency peaks to calculate the variations of the degenerate mode shapes. The laser spot is focused in an area of the membrane that enables detecting the five resonances with similar amplitudes (Supporting Information Section S2). An optical microscope image of the membrane sensor is shown in Figure 2a.

The dimensions of the membrane were chosen to maximize the capture area while keeping enough sensitivity to access the mass of the targeted analytes with enough precision. A complete description of the membrane specifications can be found in the Supporting Information Section S2. Figure 2b–f shows the five mode shapes of the mechanical modes of the sensor used in this work that correspond to the mode (1,1) and the two pairs of degenerate modes (1,2) and (1,3). We did not use the mode (2,2) because it could not be efficiently excited in our experimental conditions. The eigenmodes were measured using a DHM (Supporting Information Section S3). Note that, as an additional advantage, this kind of structure supports many mechanical modes resonating at close frequencies, which enables optimizing the setup for a relatively narrow frequency band. Figure 2g shows the mechanical spectra of the sensor, measured at low vacuum (0.1 mbar). Note that while the mode (1,1) is isolated in the frequency spectra, the pair (1,2) as well as the pair (1,3) resonate at very close frequencies (indicating a degeneration state) that can be identified due to the high-quality factors of these modes. Once characterized the initial state of our sensor, we deposit *E. coli* K-12 bacteria onto it while tracking the resonance frequencies and amplitudes of the five modes shown in Figure 2. The details of the preparation of the samples are described in the Supporting Information Section S4. The tracking is performed using a phase-locked loop system provided by a frequency lock-in amplifier. We used an acquisition time of 140 ms, which provides relative frequency stability below 1 ppm for all the modes measured and allows distinguishing between close events occurring in a time interval larger than 1 s (Supporting Information Section S2).

## Results and Discussion

**Figure 3:**
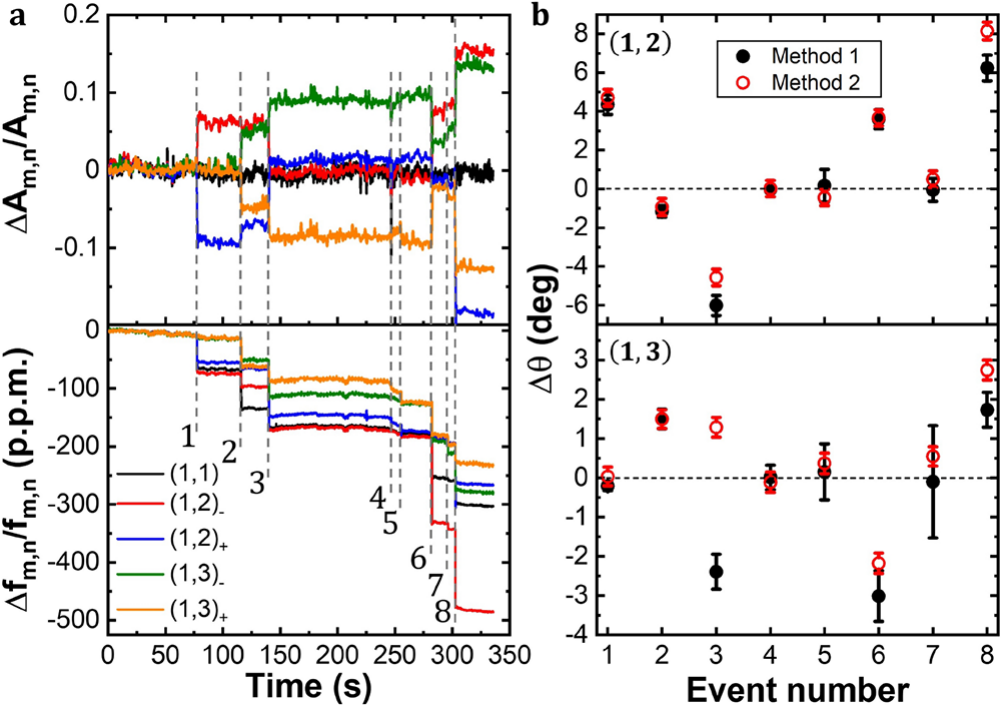
Change of amplitudes, resonance frequencies, and parameter θ during the experiment with bacteria. (a) Relative change in amplitude (top) and in frequency (bottom) for the five modes measured in the experiment. Each abrupt change corresponds to the adsorption of a bacterium. (b) Change of the parameter θ after each of the adsorption events for the pair (1,2) (top) and for the pair (1,3) (bottom) calculated by the two methods presented in this work.

Figure 3a (top and bottom) shows the relative changes in amplitudes and frequencies for the five modes measured during the experiment, respectively, where eight different events (abrupt shifts) corresponding to bacteria adsorptions can be observed.

**Figure 4:**
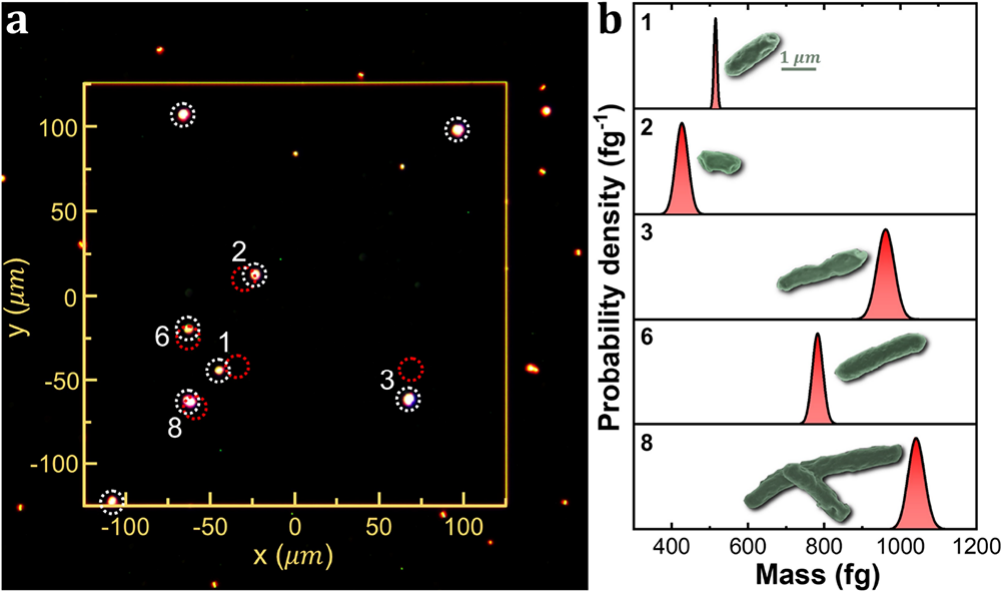
Mass distributions and positions of the bacteria calculated by the inverse problem method. (a) Dark-field image of the membrane resonator after the experiment. Each bacterial cell has been identified by SEM (white dashed circles). The positions obtained by the inverse problem are represented with red dashed circles and the numbers represent the event number chronologically ordered. We only show five events because the rest have very large errors in position. These large errors are caused by the real positions of the adsorbed bacteria that are too close to the edges of the membrane. (b) Mass distributions obtained by the inverse problem method and SEM image of each of the five bacteria shown in panel (a).

For every event, we solve the inverse problem and obtain the mass and position of each bacterium. Then, we calculate the variation Δθ caused by the adsorption using the two methods presented above and update the mode shapes for the next jump. Figure 3b (top and bottom) shows the variation of the mode shapes Δθ for the pair (1,2) and for the pair (1,3), respectively, calculated by the two different methods. We can see that the values obtained by both methods match reasonably well within the error intervals. Just the third event for the pair (1,3) presents an important deviation between the two methods. The signal-to-noise ratio is higher for the amplitude changes compared to frequency changes, as it can be observed in Figure 3a. However, the error in the first method is, in general, higher than the error obtained with the second method. The reason is that for the method that uses the frequency changes, two steps are needed to calculate Δθ. The first step calculates the mass and position of the adsorbed particle, and the second step calculates Δθ using eq 11. Therefore, the errors propagate from the first step to the second. In contrast, the method that uses the amplitude changes involves just one step in the calculation of Δθ, making it, in general, more accurate. It can be observed that some events barely change the mode shapes but some others can produce important changes, of the order of 8 degrees. Obviously, the change of the mode shapes depends on the mass and adsorption position of the bacteria. Figure 4a shows a dark-field microscope image of the membrane after the experiment, where all the deposited bacteria can be clearly identified. The adsorption positions indicated in the image have been obtained by applying the inverse problem and using the method of the change in the amplitudes (method 2) to update the mode shapes after each event. We can see that all the bacteria that are far from the edges of the membrane have been well located by the inverse problem. However, those cells that have been adsorbed closer to the edges present higher deviation with respect to the true position. This is because, close to the edges of the membrane, where the amplitude of the vibration is smaller, the mass effect decreases, causing a degradation of the precision. Figure 4b shows the mass probability density function of the five individual bacterial cells calculated by the inverse problem as well as a SEM image of each of the identified bacteria.

As stated in the theoretical section, the main parameters that will determine the magnitude of the changes of the mode shape are the ratio between the mass of the analyte to the mass of the membrane and the separation of the two frequencies associated to a pair of degenerate modes (Δ_0_). This last parameter depends strongly on the aspect ratio of the membrane, and therefore, one way to avoid the effects of mode degeneration is to choose a higher value for the aspect ratio of the membrane sensor at the cost of reducing the capture efficiency. This will lead, in general, to better accuracy in the calculation of the mass and position of the adsorbed particles, since there will be no errors associated to the process of updating the mode shapes. Another aspect is that although the new theory solves the problem of mode degeneration for nanomechanical MS using bi-axisymmetric membranes as the sensor element, the mathematical expressions obtained are more complex, increasing the computational time. Therefore, depending on the particular necessity, one might want to use a higher aspect ratio to gain computational time and accuracy at the cost of losing some capture efficiency, or if the most important aspect is the capture efficiency, we can use an aspect ratio closer to 1 and use the degeneration theory at the cost of more computational time and less accuracy.

**Figure 5:**
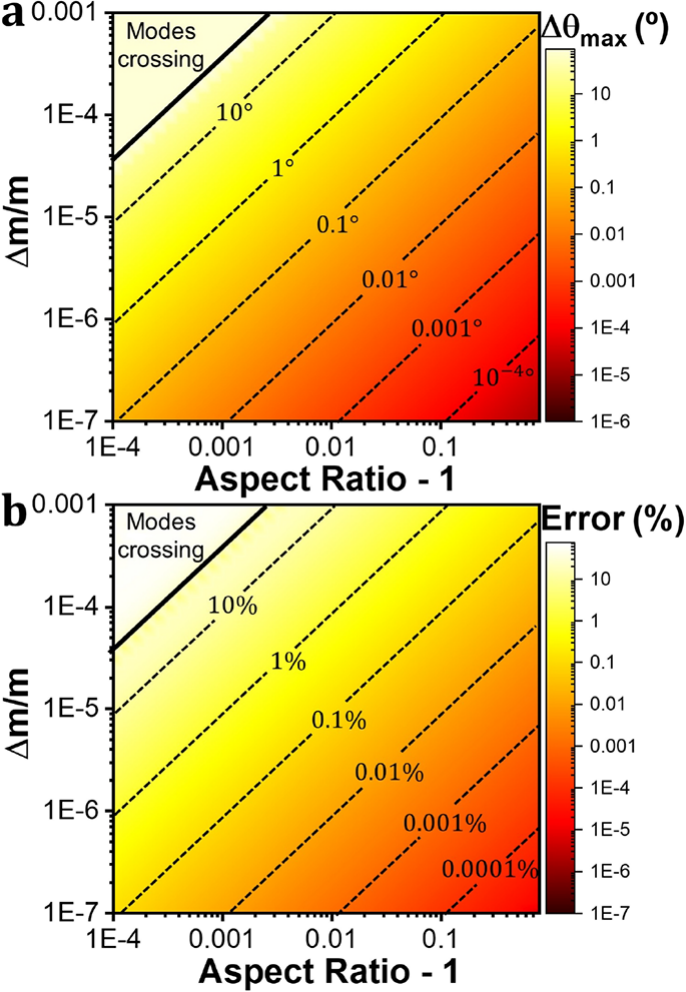
Maximum change in θ and error in the relative frequency shift. (a) Maximum change in θ (deg) that a single particle will produce in the pair of modes (1,2) as a function of the ratio between the mass of the particle and the mass of the membrane and the aspect ratio of the membrane. (b) Error in the calculation of the relative frequency shift relative to the maximum value −2Δm/m for the pair of modes (1,2) neglecting the mode change and using eq 13 as a function of the ratio between the mass of the particle and the mass of the membrane and the aspect ratio of the membrane.

Figure 5a shows the maximum change in the mode shape for the pair of modes (1,2) that will be produced by an individual adsorption as a function of the aspect ratio of the membrane and the ratio between the mass of the analyte and the mass of the membrane. Figure 5b shows the maximum error that will be committed in the calculation of the relative frequency shift (normalized to the maximum relative frequency shift −2Δ*m*/*m*) neglecting the effects of degeneration for the pair of modes (1,2), i.e., using eq 13 instead of eq 12, as a function of the aspect ratio of the membrane and the ratio between the mass of the analyte and the mass of the sensor. The continuous black line in both graphs represents a change in θ of 90° (modes crossing). Obviously, an error in the determination of the frequency shift will lead to errors in the calculation of the mass and adsorption position. Very similar results can be obtained for the pair of modes (1,3) (Supporting Information Section S5).

Of course, for a long experiment in which hundreds of analytes are expected to reach the sensor, the separation of the frequencies will vary as a random walk throughout the whole experiment, and we can get to a point where this separation becomes so small that the changes of the mode shapes start to be important. Therefore, even if the initial conditions of the experiment meet the required conditions to avoid degeneration effects, the frequency separation should be always considered to avoid non-desirable effects.

## Conclusions

In this work, we have investigated the assets of membrane mechanical resonators when used as sensors for nanomechanical mass spectrometers. In principle, they are ideally suited for it due to their large capture areas and reduced thicknesses as they achieve high capture efficiency while keeping enough sensitivity. However, here, we have showed that if their aspect ratio is very close to 1, they support degenerate modes that are prompt to changes in their shapes when particles are adsorbed. This effect significantly complicates the interpretation of the sensor response and is critical for the correct calculation of the mass and position of the adsorbed particles. We have developed a novel theoretical model that precisely predicts the response of the sensors to particle adsorptions that accounts for mode degeneration, and we have validated it through numerical simulations. Based on this new model, we have proposed two different methods to update the mode shapes right after an adsorption event occurs, one based on direct calculation of the mass and position of the adsorbed particle and another one based on the change of the amplitudes, in which our results indicate to be more precise. Finally, we have validated the theory and the two methods for eigenmode change calculation in an experiment depositing several *E. coli* bacterial cells on a square silicon nitride membrane resonator. We obtained their adsorption positions and mass distributions as well as the change of the mode shapes after every adsorption event, proving that the presented new theory allows the use of high-capture-efficiency resonators that support degenerate modes in nanomechanical MS.

## Supporting Information Available

Details about the membranes’ specifications, frequency tracking setup, and biological sample preparation (PDF)
