# Supplementary material for: Square Membrane Resonators Supporting Degenerate Modes of Vibration for High-Throughput Mass Spectrometry of Single Bacterial Cells
Source: ACS Sens. 2023 May 1;8(5):2060–7. doi: 10.1021/acssensors.3c00338 (PMC10226162; doi:10.1021/acssensors.3c00338)
Supplement: Supplementary file 1 — se3c00338_si_001.pdf [file se3c00338_si_001.pdf]

Supporting information

Square membrane resonators supporting degenerate modes of vibration for high-throughput mass spectrometry of single bacterial cells

Adrián Sanz-Jiménez, Jose J. Ruz*, Eduardo Gil-Santos, Oscar Malvar, Sergio García-López, Priscila M. Kosaka, Álvaro Cano, Montserrat Calleja and Javier Tamayo

Bionanomechanics Lab, Instituto de Micro y Nanotecnología, IMN-CNM (CSIC), Isaac Newton 8 (PTM), E-28760 Tres Cantos, Madrid, Spain

Section S1: Finite element simulations for different mass ratios and range of validity of the theory

We have performed finite element simulations of the adsorption of a point-like particle on a stressed silicon nitride membrane of 50 nm thickness, 250 μm width, aspect ratio of 1.0001 and a total mass of 9.6875 ng. A pre-stress of 212 MPa is applied to the membrane before the eigenfrequency analysis. The position of adsorption is $(X_0,X_0) = (-0.3, -0.2)$. This position has been chosen because the effect of degeneration is particularly high if the original mode shapes are those corresponding to the non-degenerate state. We varied the mass of the particle so that the mass ratio takes values from 10^{-7} to 10^{-2}. We fit the mode shapes obtained in the simulations to equation (10) in the main text and calculate the R^2 coefficient in each case in order to see if the theory can still describe the mode shapes correctly (Figure S1a). We also obtained the value of the parameter θ for the pair (1,2) and for the pair (1,3) and compared the results with the values predicted by the theory (Figure S1b). It can be seen in figure S1 that the coefficient R^2 is very close to one for all the modes and it only degrades for values higher than 10^{-3} where the value starts to drop quickly. Therefore, we can say that this is the limit of validation of our theory before other effects start to be relevant. Interestingly, the coefficient R^2 only degrades for the modes $(1,2)_-$ and $(1,3)_-$. The reason is that, due to degeneration, when the mass of the adsorbed particle is very high, the change in the parameter θ can be so high that one of the modes of a degenerate couple changes its nodal lines to make it pass through the adsorption position. For this particular adsorption position, those modes happen to be the modes $(1,2)_+$ and $(1,3)_+$. If there is a nodal line at the adsorption position, there is no mass effect and therefore the mode remains unperturbed.

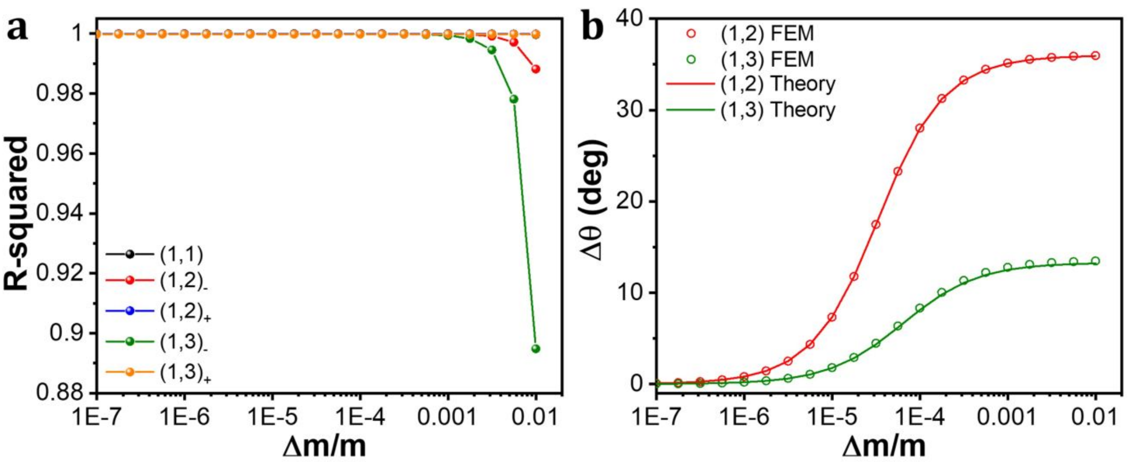

Figure S1 | *Finite element simulations of the adsorption of a particle on the membrane varying the mass ratio between the particle and the membrane. a) Coefficient R^2 obtained from fitting the mode shapes obtained by the simulations to equation (10) in the main text. Modes (1,1), $(1,2)_+$ and $(1,3)_+$ are very close to one for all the mass ratio range and are superposed. b) Values of parameter θ obtained by the simulations compared to the expected by the theory as a function of the mass ratio for the modes (1,2) and (1,3).*

Section S2: Membrane specifications and monitorization of the resonance frequencies by a laser beam deflection setup

The membrane resonator used in the experiments was fabricated by Norcada Inc. It has a nominal thickness of (54 ± 2) nm and its planar dimensions were accurately characterized through scanning electron microscope (SEM) images giving a length of (258.5 ± 0.5) µm and a width of (258.3 ± 0.5) µm. The membrane is made out of low stress silicon nitride, and due to the fabrication process, it has a nominal stress of (212 ± 15) MPa. The size of the chip that contains the membrane is $5\ mm \times 5\ mm\ (\pm 0.2\ mm)$ and it is a stacking of three layers: two silicon nitride layers, in top and bottom, and a silicon layer in between. The nominal thicknesses of the silicon nitride layers are $(50 \pm 2)\ nm$, while the nominal thickness of the silicon layer is $(200 \pm 5)\ \mu m$.

The mechanical spectra of the membrane resonator were measured by means of the laser beam deflection technique[1,2] and using a lock-in amplifier from Zurich Instruments Ltd. (HF2LI 50 MHz Lock-in Amplifier). The laser used in the experiments was manufactured by Schäfter + Kirchhoff GmbH (13MC-M60+95CM-635-10-B21-M60-C6). Its wavelength is $639\ nm$, the output power was set at $550\ \mu W$ and the beam reaches the membrane with an inclination of $30°$. The light collected from the reflection in the membrane was focused by a 10x magnification objective from Mitutoyo Europe GmbH (M Plan APO 10x) in a quadrant Si PIN photodiode (S4349 from Hamamatsu Photonics K. K.). Notably, in our configuration, the signal amplitude is proportional to the slope of the out-of-plane displacement in the x-direction[1]. In order to optimize the measurement of all the frequencies at the same time, we focused the laser spot around $x = 0$ and $y \cong 0.40 L_y$, which corresponds to a point where the absolute value of the product of all the slopes in the x-direction is maximum. Figures S2a-c show the resonances (closed circles) and the phase delays (open circles) of the modes used in the experiments, measured by the lock-in amplifier. In order to make sure that we do not reach non-linear regime, we excite the resonators with a piezo shaker with very small voltage. We measure the frequency peak and the phase delay. Then, we increase the applied voltage until the Allan variance is optimum making

sure that the phase remains unchanged and no duffing effects are observed. The resonance frequencies of the modes of Figure S2 and their Q-factors are: $f_{(1,1)} = 735.2\ kHz$ and $Q_{(1,1)} = 2300$, $f_{(1,2)_-} = 1162.1\ kHz$ and $Q_{(1,2)_-} = 4700$, $f_{(1,2)_+} = 1163.0\ kHz$ and $Q_{(1,2)_+} = 4650$, $f_{(1,3)_-} = 1643.3\ kHz$ and $Q_{(1,3)_-} = 5900$ and $f_{(1,3)_+} = 1644.9\ kHz$ and $Q_{(1,3)_+} = 5900$. Along the bacteria adsorption experiments, the mechanical frequencies of these modes were tracked simultaneously using a multimode phase-locked loop (PLL) system, provided by the lock-in amplifier.

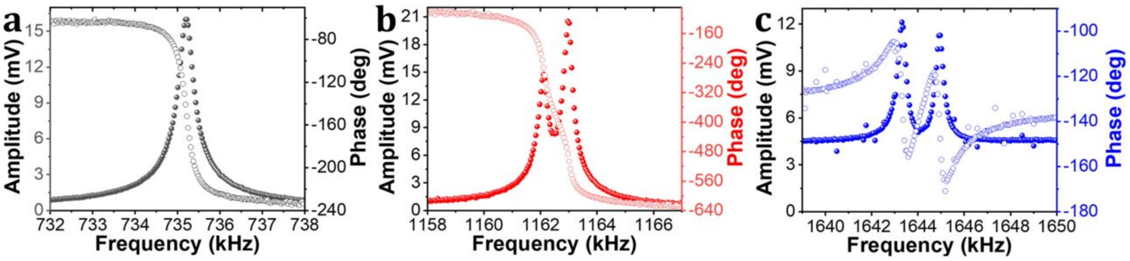

Figure S2 | *Amplitude (closed circles) and phase response (open circles) of the membrane resonator around the frequencies of the vibration modes used in the experiments. a) Non-degenerate* $(1,1)$ *mode. b) Degenerate pair* $(1,2)_-$ *and* $(1,2)_+$ *modes. c) Degenerate pair* $(1,3)_-$ *and* $(1,3)_+$ *modes.*

Figure S3 shows the Allan standard deviations of the resonance frequencies associated to the previously shown mechanical modes. These measurements were performed in closed-loop configurations. Proportional, integral and derivative parameters (PID parameters) from the PLL system were selectively chosen to obtain a behaviour of the different frequencies similar to that of open loop. In Figure S3, it can be seen that the frequency sensitivity degrades for larger acquisition times for every vibration mode, due to the thermal drift[1]. Therefore, in the bacteria adsorption experiments, we set the acquisition time to 0.14 s, as it is the time that presents lower noise for every mode. At this acquisition time, the corresponding relative frequency noise for the five vibration modes of the experiments, $(1,1)$, $(1,2)_-$, $(1,2)_+$, $(1,3)_-$ and $(1,3)_+$, are $3.7 \cdot 10^{-7}$, $3.6 \cdot 10^{-7}$, $3.9 \cdot 10^{-7}$, $7.1 \cdot 10^{-7}$ and $7.4 \cdot 10^{-7}$, respectively.

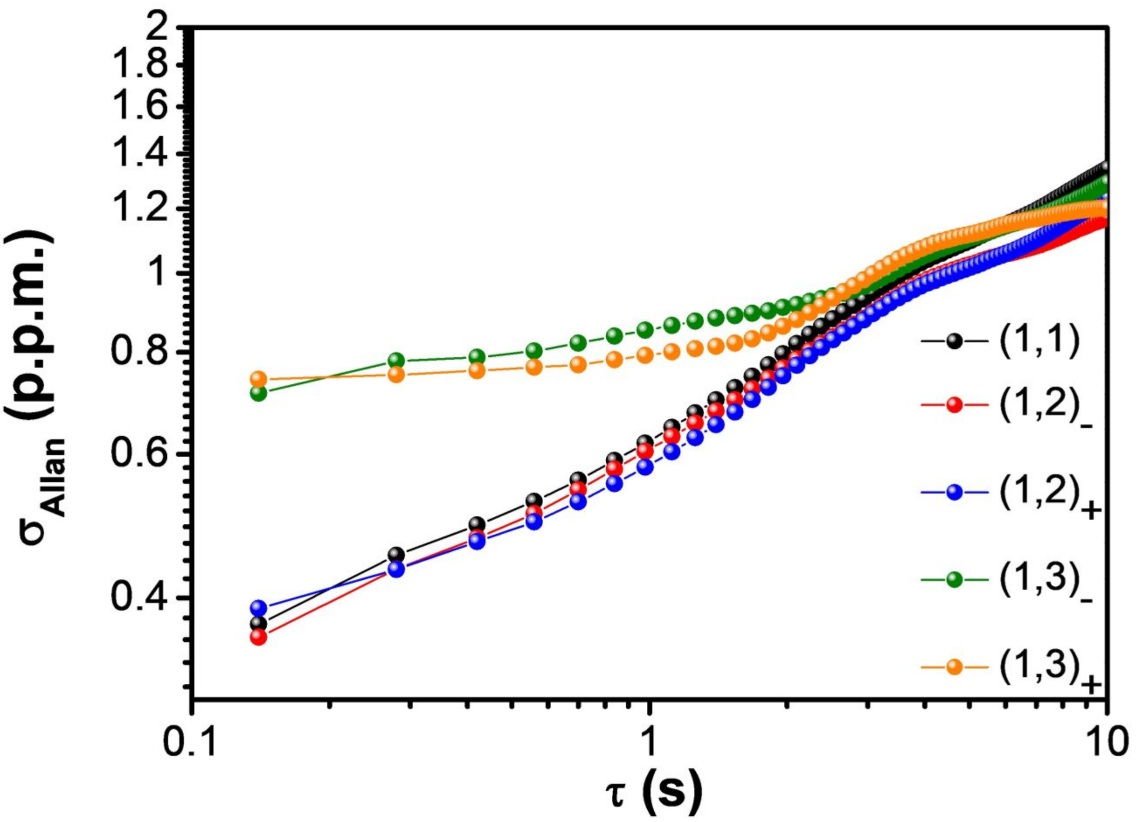

Figure S3 | *Allan standard deviations of the resonance frequencies of the vibration modes used in the experiments measured in closed-loop configuration.*

Section S3: Imaging the membrane vibration modes using a digital holographic microscope (DHM)

Figure S4 shows a schematic depiction of the setup used for the measurements of the mode shape profiles of Figure 2b-2f of the main text. The membrane resonator was placed in a home-made vacuum system at a pressure of about $10^{-3}\ torr$ and at room temperature. The vacuum was kept by a dual-stage rotary vane vacuum pump (TRIVAC E2 D2.5E from Oerlikon Leybold Vacuum GmbH). The mechanical modes were driven by a piezoelectric (miCos Iberia S. L.) located underneath the resonator. Both, the membrane resonator and the piezoelectric, were mounted above a 3D micro-positioner stage (Sigmakoki CO., LTD.) that enables their precise location in the three spatial directions. The DHM used is a reflection single laser source R1000 from Lyncée Tec. The objective used was a 10x magnification objective lens from Leica Camera AG. The MEMS Analysis Tool software of Lyncée Tec was used to obtain the mode profiles after recording the phase delay of eight oscillation cycles of the resonance frequencies[1].

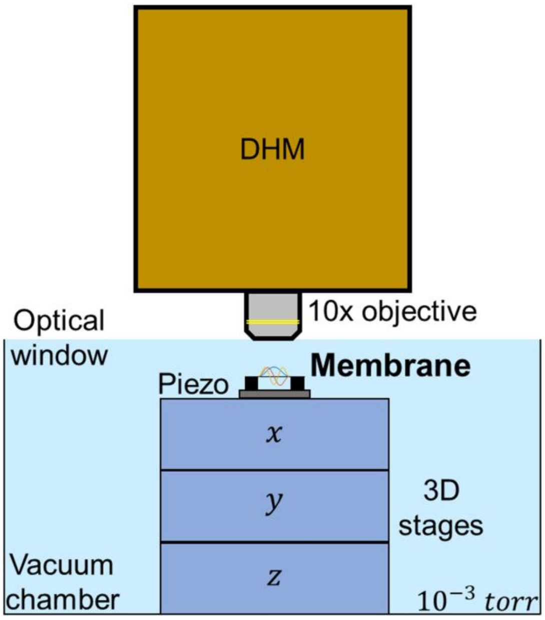

Figure S4 | *Schematic depiction of the home-made setup employed for measuring the membrane mechanical mode shapes, based on the use of a digital holographic microscope (DHM). The membrane resonator is placed inside a vacuum chamber kept at room temperature, which reaches a pressure of about 10^{-3} $torr$. A piezoelectric is placed below the membrane in order to actuate the vibration modes. Both, the piezoelectric and the membrane, are placed above a 3D micro-positioner stage that enables their precise location in the three spatial directions.*

Section S4: Experiments with bacterial cells using the nanomechanical spectrometer

The bacteria used in the experiments are *Escherichia coli* K-12, kindly supplied by Dr. Jesús Mingorance from Microbiology Department of Hospital La Paz (Madrid, Spain). The preparation consisted in adding 10 mL of Luria-Bertani broth (Sigma-Aldrich, USA) to $50\ \mu L$ of a stationary phase culture. The *E. coli* bacteria were grown overnight at 37^0C under agitation. Then, the bacteria were centrifugated at 3500 rpm during 5 min at 20^0C and resuspended in Mili-Q water. After three times, the bacteria were resuspended in 50% isopropyl alcohol/Mili-Q water. The final concentration was about $3 \times 10^9 mL^{-1}$. The solution of bacteria was loaded in a $250\ \mu L$ glass syringe pump (CETONI GmbH). A constant flow rate of $0.3\ \mu L/min$ was set by a syringe pump flow controller (Base 120, CETONI GmbH) during the experiments. An electrospray ionization (ESI) source designed and manufactured by Fasmatech Science and Technology S.A., was used to spray the solution. The high voltage difference between the metal ESI needle from Thermo Fisher Scientific, S. L. U. (inner diameter of $51\ \mu m$) and the nanomechanical spectrometer entrance was set to $2-3\ kV$. The high voltage was supplied by a high voltage bipolar power supply (HV 7010, Ioner Aerosol & Volatile Analysis Technology), and the ESI needle was separated by about $10\ mm$ from the nanomechanical spectrometer entrance, in order to avoid droplets in the samples. Curtain gas and sheath gas were used to prevent big particles and solvent droplets to enter the spectrometer. The flow of both gases was set to $300\ mL/min$. At the entrance of the nanomechanical spectrometer, a capillary inlet with $500\ \mu m$ of inner diameter was placed and heated at about 150 ℃, in order to ensure solvent evaporation. Further details about the nanomechanical spectrometer used in the experiments are explained elsewhere[1].

Section S5: Change of the mode shape and maximum error committed in the relative frequency shift for the pair of modes (1,3)

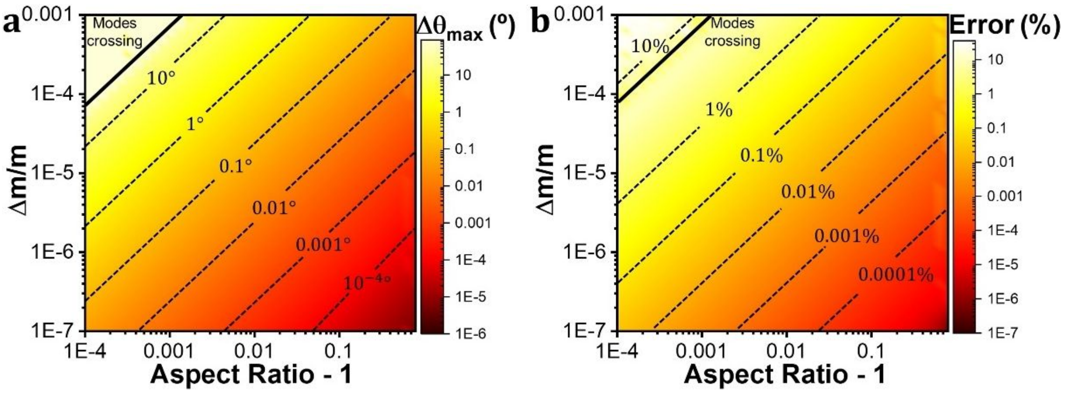

Figure S5 | Maximum change in θ and error in the relative frequency shift. (a) *Maximum change in θ (deg) that a single particle will produce in the pair of modes (1,3) as a function of the ratio between the mass of the particle and the mass of the membrane and the aspect ratio of the membrane.* ***(b)*** *Error in the calculation of the relative frequency shift relative to the maximum value $-2\Delta m/m$ for the pair of modes (1,3) neglecting the modes change and using equation (13) of the main text as a function of the ratio between the mass of the particle and the mass of the membrane and the aspect ratio of the membrane.*

References

(1) Sanz-Jiménez, A.; Malvar, O.; Ruz, J. J.; García-López, S.; Kosaka, P. M.; Gil-Santos, E.; Cano, Á.; Papanastasiou, D.; Kounadis, D.; Mingorance, J.; Paulo, Á. S.; Calleja, M.; Tamayo, J. High-Throughput Determination of Dry Mass of Single Bacterial Cells by Ultrathin Membrane Resonators. *Commun. Biol.* **2022**, *5* (1), 1227.

(2) Tamayo, J.; Pini, V.; Kosaka, P.; Martinez, N. F.; Ahumada, O.; Calleja, M. Imaging the Surface Stress and Vibration Modes of a Microcantilever by Laser Beam Deflection Microscopy. *Nanotechnology* **2012**, *23* (31), 315501.
